# One-Vat Multimaterial 3D Printing: The Devil is in the Details

**DOI:** 10.1021/acscentsci.5c00986

**Published:** 2025-08-07

**Authors:** Elizabeth A. Recker, Xiaofeng Chen, Ji-Won Kim, Zachariah A. Page

**Affiliations:** † McKetta Department of Chemical Engineering, 12330 The University of Texas at Austin, Austin, Texas 78712, United States; ‡ Department of Chemistry, 12330 The University of Texas at Austin, Austin, Texas 78712, United States

## Abstract

Nature combines different materials in a single structure to achieve functions that no single material could accomplish alone, an approach that inspires efforts to build synthetic systems with precisely tailored properties. Vat photopolymerization (VPP) enables fast, high-resolution 3D printing, but most printed parts still use only one material. This Outlook highlights emerging strategies for single-vat multimaterial VPP, where light selectively activates different chemical reactions to build complex structures with multiple materials. Key advances will depend on expanding resin chemistry beyond standard acrylates, improving reaction selectivity, and using grayscale and multiwavelength light control to define where and how materials form. Standardized mechanical, thermal, and interface testing methods are essential for ensuring reliable results. With advances in chemistry, optics, and data-driven design, multimaterial VPP could unlock transformative applications in medicine, manufacturing, and aerospace.

## Graphical abstract

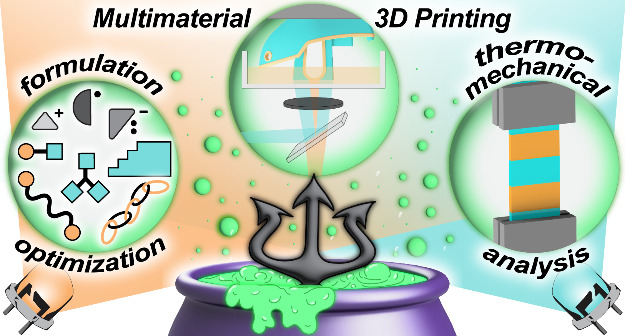

## 1 Introduction

Biological systems exemplify the power of multimaterial integration, combining diverse components, such as soft and hard tissues, into structures with exceptional, multifunctional performance. Inspired by this synergy, researchers are developing synthetic systems with spatially tailored properties to meet needs in healthcare, transportation, and beyond.^1^ Among additive manufacturing methods, vat photopolymerization (VPP) offers a unique combination of speed, precision, and scalability, yet most commercial systems produce single-material, monolithic objects.^2^ Goals for advancing multimaterial VPP include improved resolution, broader material scope, spatial control, part stability, and reliable interfaces—all while minimizing cost.^3^ While topology optimization has enabled monolithic structures with tunable stiffness through geometric design, it remains limited in property contrast and design flexibility.^4^ In comparison, multimaterial printing enables functional variation across mechanical,^5^ thermal,^6^ optical,^7^ and electrical^8^ properties, with greater design freedom.

This Outlook presents strategies for translational multimaterial VPP, focusing on “one-pot” systems that use selective chemical activation to create heterogeneous structures without compromising speed or resolution (Figure 1). Organized chronologically, we explore three key areas:

**Figure 1:**
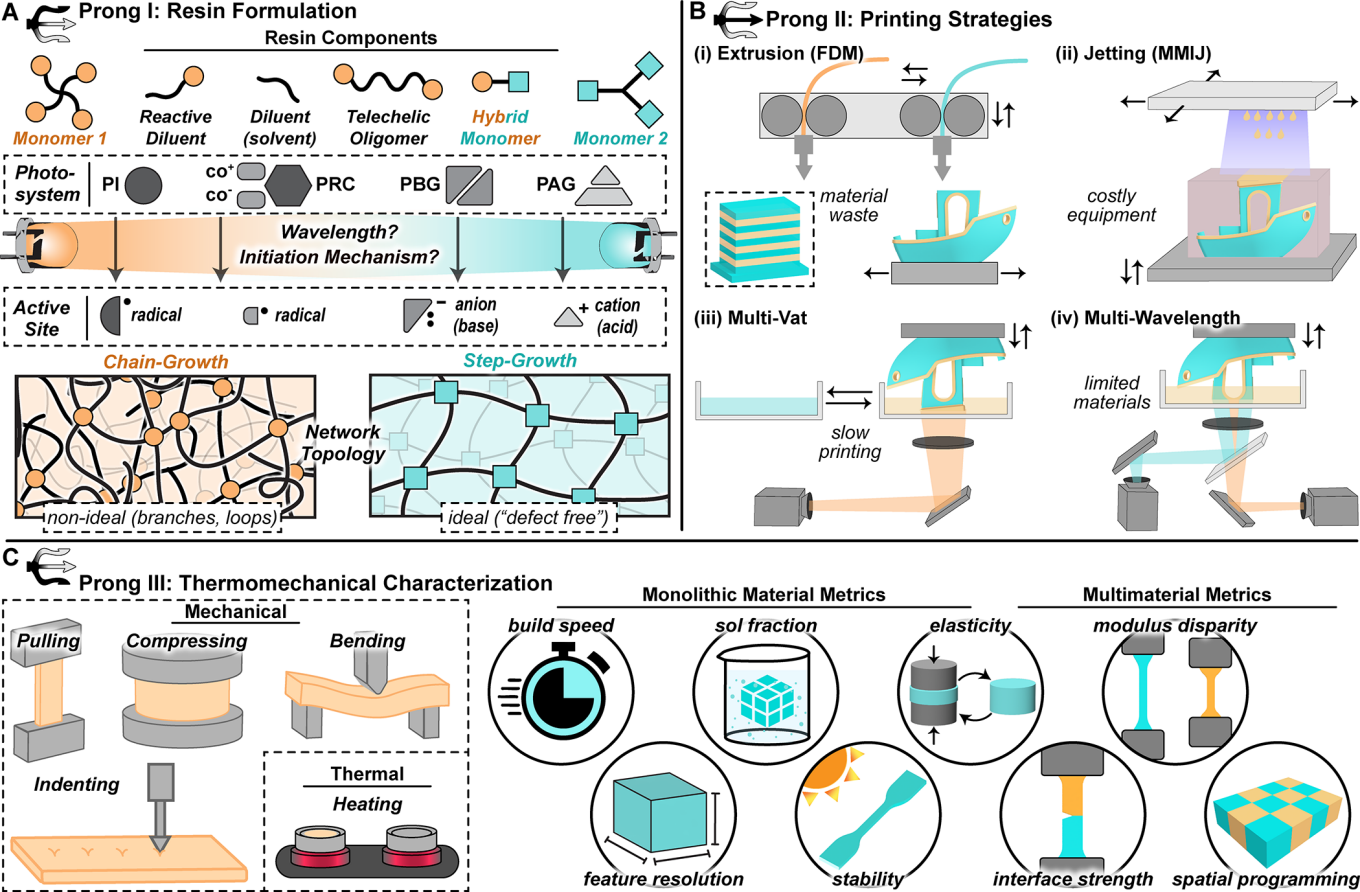
Key stages in multimaterial 3D printing. (A) Light-activated monomers undergo either chain or step growth to form polymer networks with distinct topologies. Abbreviations: PI, photoinitiator; PRC, photoredox catalyst; PAG, photoacid generator; PBG, photobase generator. (B) Multimaterial fabrication approaches include extrusion (FDM), ink jetting (MMIJ), and vat photopolymerization (VPP) using multivat or multiwavelength configurations. (C) Monolithic and multimaterial constructs are evaluated via tensile, compression, bending, nanoindentation, and calorimetry techniques, with metrics including resolution, sol fraction, elasticity, interface strength, modulus disparity, and spatial programming.

### Prong I: Resin Formulation

Photopolymerization uses light to solidify resins through diverse initiation mechanisms (e.g., one- and two-electron processes), leading to network structures with distinct molecular heterogeneity (Figure 1A). Understanding these pathways is essential for designing resins with targeted mechanical properties.^9^

### Prong II: Printing Strategies

The most accessible methods for 3D printing are extrusion-based techniques (e.g., fused deposition modeling (FDM), multimaterial inkjet printing (MMIJ)), but they suffer from low resolution and weak intermaterial bonding. On the other end of the spectrum, multiphoton polymerization (MPP) enables submicron resolution, but it remains limited by low throughput, high equipment costs, and poor scalability.^10,11^ One avenue toward multimaterial 3D printing is multimodality printers, which combine multiple 3D printing techniques into a single system; however, this approach requires highly specialized hardware and custom software integration.^12,13^ Alternatively, VPP methods like digital light processing (DLP) offer superior resolution and speed (Figure 1B).^14^ However, current multimaterial VPP is largely multivat based, which slows printing and risks cross-contamination.^15^ One-pot systems, leveraging multiwavelength or grayscale light projection, show promise for multimaterial fabrication but still face challenges in resolution, throughput, and property contrast.^9,16–26^

### Prong III: Thermomechanical Characterization

Assessing key properties, such as stiffness, strength, toughness, elasticity, and glass transition temperature, using standardized methods is essential for ensuring consistency and reproducibility (Figure 1C). Opportunities in one-pot multimaterial systems include reducing sol fractions, enhancing photothermal stability, increasing material contrast, and enabling spatially graded properties. At the same time, material systems must be carefully tailored to meet the specific performance demands of their target applications.

Realizing the potential of multimaterial VPP will require rigorous practices across formulation, printing, postprocessing, and characterization. Transparent reporting of material properties is essential for meaningful comparisons, and reproducibility should be supported by statistically significant sample sizes (e.g., ≥5 samples for mechanical testing) and a coefficient of variation below 5%.

## 2 Prongs of Multimaterial VPP 3D Printing

### 2.1 Prong I: Resin Formulation

*How can the research community unlock new reaction pathways and enhance selectivity to broaden the chemical scope of one-pot multimaterial VPP?*

Light attenuation from absorption and scattering can lead to uneven curing, especially in systems that use multiple wavelengths. Photobleaching and opacification can alter resin transparency, affecting cure depth and kinetics, highlighting the need for real-time optical monitoring. Polymerization kinetics must balance fast initiation with efficient termination, yet managing distinct kinetic profiles in multimaterial systems remains an open challenge. Oxygen inhibition improves contrast but may undercure fine features, with added complexity in multimaterial resins due to varying oxygen sensitivity. Finally, one-pot multimaterial printing demands careful tuning of the wavelength and dosage to achieve selective and cooperative reactivity across distinct material domains. Multiwavelength printing can operate through four modes: (i) *Constructive*—multiple wavelengths activate a reaction; (ii) *Destructive*—individual wavelengths react but jointly inhibit; (iii) *Orthogonal*—distinct wavelengths trigger separate reactions; (iv) *Dominant*—one wavelength is general, the other selective (Figure 2A). In considering these points, advancing one-pot multimaterial VPP will require a deeper photochemical characterization of resins (Section 2.1.1) and an expansion of resin chemistries (Section 2.1.2).

Rapid, high-resolution multimaterial VPP relies on precise control of light–matter interactions, grounded in a systematic understanding of resin photochemistry.

**Figure 2:**
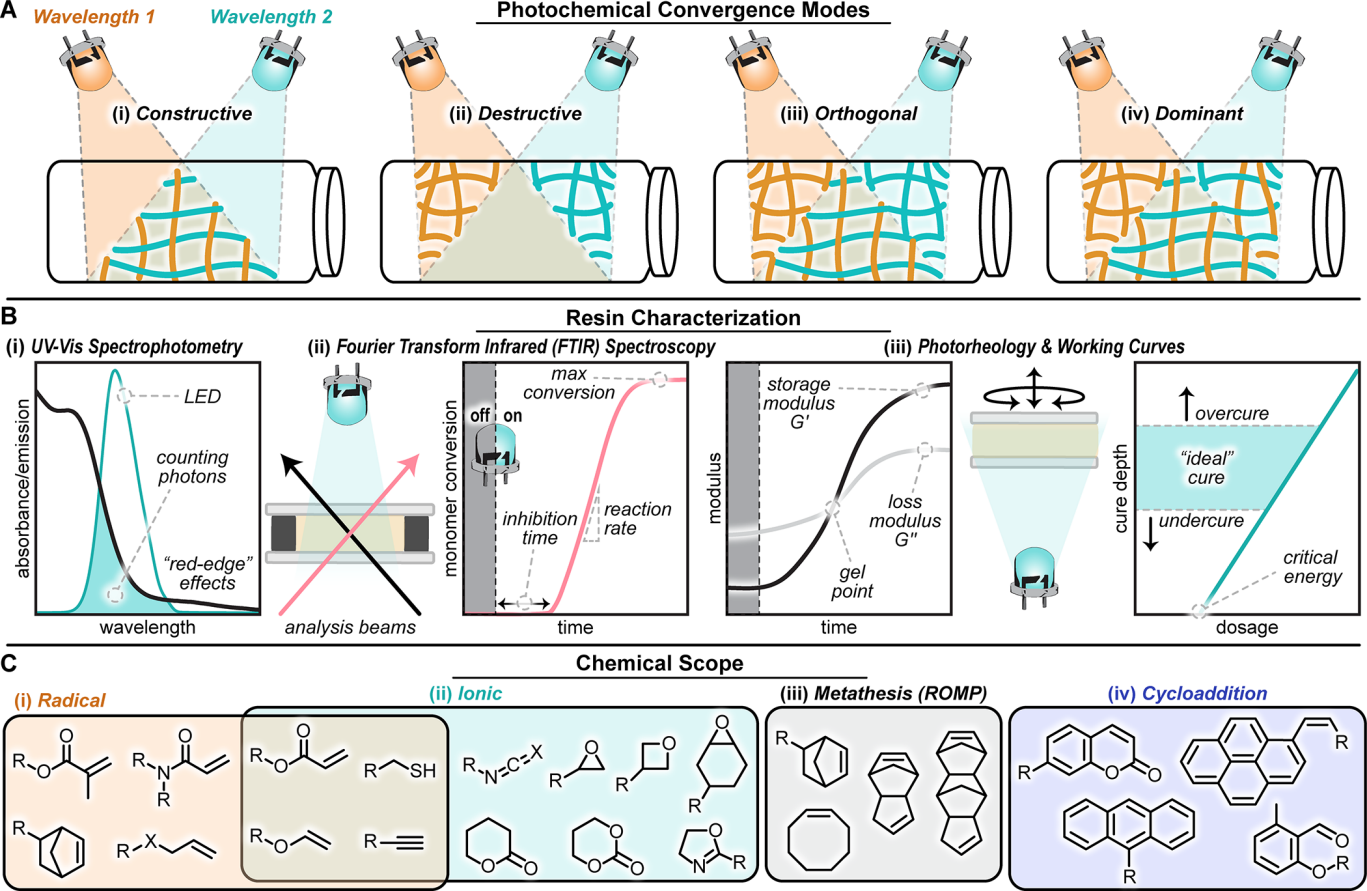
Design considerations for multimaterial resin systems. (A) Selective activation of distinct reactions can be achieved through constructive, destructive, orthogonal, or dominant convergence of multiple light sources. (B) Techniques such as UV–vis absorption and FTIR spectroscopy and photorheology are used to evaluate photon absorption, conversion efficiency, gelation behavior, and cure depth, key parameters for optimizing print conditions. (C) A range of photopolymerization mechanisms, radical, ionic, ring-opening metathesis, and photoinduced cycloadditions, have the potential for integration into one-pot multimaterial VPP.

#### 2.1.1 Resin Characterization

The optical properties of resins play a key role in controlling the photopolymerization kinetics and cure depth in VPP 3D printing (Figure 2B). Characterization begins with UV–visible spectrophotometry to map absorbance profiles and assess overlaps with light source emissions (Figure 2B,i). Though marketed as “monochromatic”, LEDs typically have spectral bandwidths of 10–30 nm (fwhm) and vary in emission characteristics, even across nominally identical models. Since every absorbed photon can initiate polymerization, quantifying absorption is critical. Combining photometer and spectrophotometer data can quantify the photon absorption across the overlap region. However, absorption alone does not always predict the reactivity. Recent action plots revealed a “red-edge” effect—where longer wavelengths yielded higher-than-expected quantum efficiencies—indicating that lower-energy photons may be more effective in some systems.^27^ Understanding the prevalence of this effect could guide the design of wavelength-selective light sources for multimaterial VPP. Furthermore, as resin absorbance may shift during curing, in situ UV–vis spectroscopy can capture these dynamic changes and their influence on reaction kinetics.

Photopolymerization kinetics can be tracked via real-time FTIR spectroscopy^28^ and photorheology.^29^ FTIR enables tracking of reactive functional groups (e.g., alkenes, epoxides, thiols, isocyanates), allowing quantification of conversion (ρ) through integration of disappearing monomer peaks (Figure 2B,ii). From this, metrics, such as inhibition period, polymerization rate, and maximum conversion, can be extracted. Future work should focus on deconvoluting overlapping IR signatures, such as those between acrylates and epoxides, to better resolve reaction selectivity in complex resin systems. Notably, techniques like photo-DSC,^30^ photo-Raman,^31,32^ and photo-NMR^33^ spectroscopy offer complementary insights into competitive reaction pathways.

Photorheology offers real-time insight into resin mechanics during curing by tracking the transition from liquid-like (*G*″) to solid-like (*G*′) behavior under light exposure (Figure 2B,iii).^29^ The crossover point of *G*′ and *G*″ is commonly used to estimate the gelation threshold, a key metric for determining the minimum energy dose needed for voxel curing. In multimaterial systems, however, detecting distinct gelation events remains challenging. Rheology can also provide resin viscosity (typically 0.25–5 Pa·s for VPP), which affects recoating and resolution. Additionally, cure depth can be related to light dosage using working curves derived from photorheology^34^ (Figure 2B,iii), but current protocols lack standardization. In fact, a recent interlaboratory study revealed inconsistencies,^35^ and models like the Jacobs equation fail to account for higher-order effects such as changes in optical attenuation and heat generation during curing.^36^ Addressing these gaps presents an opportunity to develop more predictive methods. Notably, optical coherence tomography (OCT) shows promise for high-resolution, in situ cure depth monitoring and could serve as a future standard.^37^

#### 2.1.2 Chemical Scope

Despite growing interest in expanding the chemical diversity of VPP resins, most systems still rely on the high-energy UV-induced radical chain-growth polymerization of acrylates (Figure 2C). Expanding beyond this paradigm has the potential to access a wider range of properties, while enabling multimaterial printing through stimuli-selective reactivity. However, alternative mechanisms remain underutilized due to challenges in curing speed, spatial control, and reaction selectivity. While one-pot multimaterial VPP has enabled spatial tuning of mechanical,^9,16,17,20–22^ optical,^23,24^ and solubility^25,26^ profiles, it remains dominated by acrylic, epoxide, and thiol chemistries (Figure 2C,i-ii). Recent studies have demonstrated the feasibility of single-material VPP using anionic polyaddition (thiol–isocyanate),^38,39^ ring-opening metathesis polymerization (ROMP)^40–42^ of strained cyclic olefins, and cationic ring-opening polymerization (ROP) of lactones,^43,44^ 2-oxazolines,^45^ and cyclic carbonates (Figure 2C,ii-iii).^46^ “Hot lithography” accelerates curing, though ionic mechanisms often have lower resolution due to limited oxygen inhibition. Critically, few photosystems combine rapid curing with wavelength selectivity, highlighting a pressing need for new photochemical platforms tailored for multimaterial printing. Complementing these efforts are one-electron (radical) mechanisms beyond acrylic chain-growth polymerization, which offer an underexplored route to functional and potentially recyclable materials.

Radical thiol–ene/yne^47–49^ and radical ring-opening^50^ polymerizations have shown promise for single-material VPP. In particular, step-growth radical mechanisms, such as nonacrylic thiol–ene reactions, offer higher monomer conversion at gelation, more uniform network structures, and lower shrinkage stress compared to chain-growth systems. These features can enhance mechanical performance and long-term part stability. Still, such systems face persistent obstacles, including poor resin pot life, slow curing kinetics, and low spatial resolution, necessitating continued advancements in resin design and printing strategies.

### 2.2 Prong II: Printing Strategies

*How can advancements in printing strategies improve both feature resolution and material fidelity in multimaterial VPP systems?*

Traditional 2D photopatterning techniques, from static masks to laser rastering and dynamic projection, have laid the foundation for multimaterial 3D printing by demonstrating spatiotemporal control and stimuli-selective activation.^51–54^ Although printing occurs downstream of resin formulation, printer limitations, such as resolution, light intensity, viscosity tolerance, and environmental control, play a central role in shaping resin design and process strategy. Advancing one-pot multimaterial VPP will therefore require coordinated improvements in the printing process (Section 2.2.1), print fidelity and calibration (Section 2.2.2), and postprint processing to preserve structural and interfacial integrity (Section 2.2.3).

Extending these capabilities into the third dimension introduces challenges in balancing print speed and resolution, achieving precise material placement, and managing complex interfacial properties across voxels.

#### 2.2.1 Printing Process

One-pot multimaterial VPP printing often leverages grayscale intensity modulation, dual-wavelength exposure, or both to achieve spatially selective reactivity. While grayscale projection enables pixel-level control of the light dose, wavelength-selective systems introduce chemically distinct domains, allowing for the fabrication of compositionally complex and functional structures (Figure 3A). Combining both approaches offers additional tunability, such as creating compositional gradients in a single print. Current digital implementations require image postprocessing, complicating timing of irradiation. These factors—simultaneous, sequential, or pulsed exposure—can affect temperature, viscosity, and ultimately, reaction selectivity. Moving forward, optimized irradiation protocols and integrated software tools that encode grayscale and wavelength-specific data prior to slicing will be essential. There is also an opportunity to develop intuitive design platforms that recommend exposure strategies based on part geometry, material requirements, and intended functionality.

**Figure 3:**
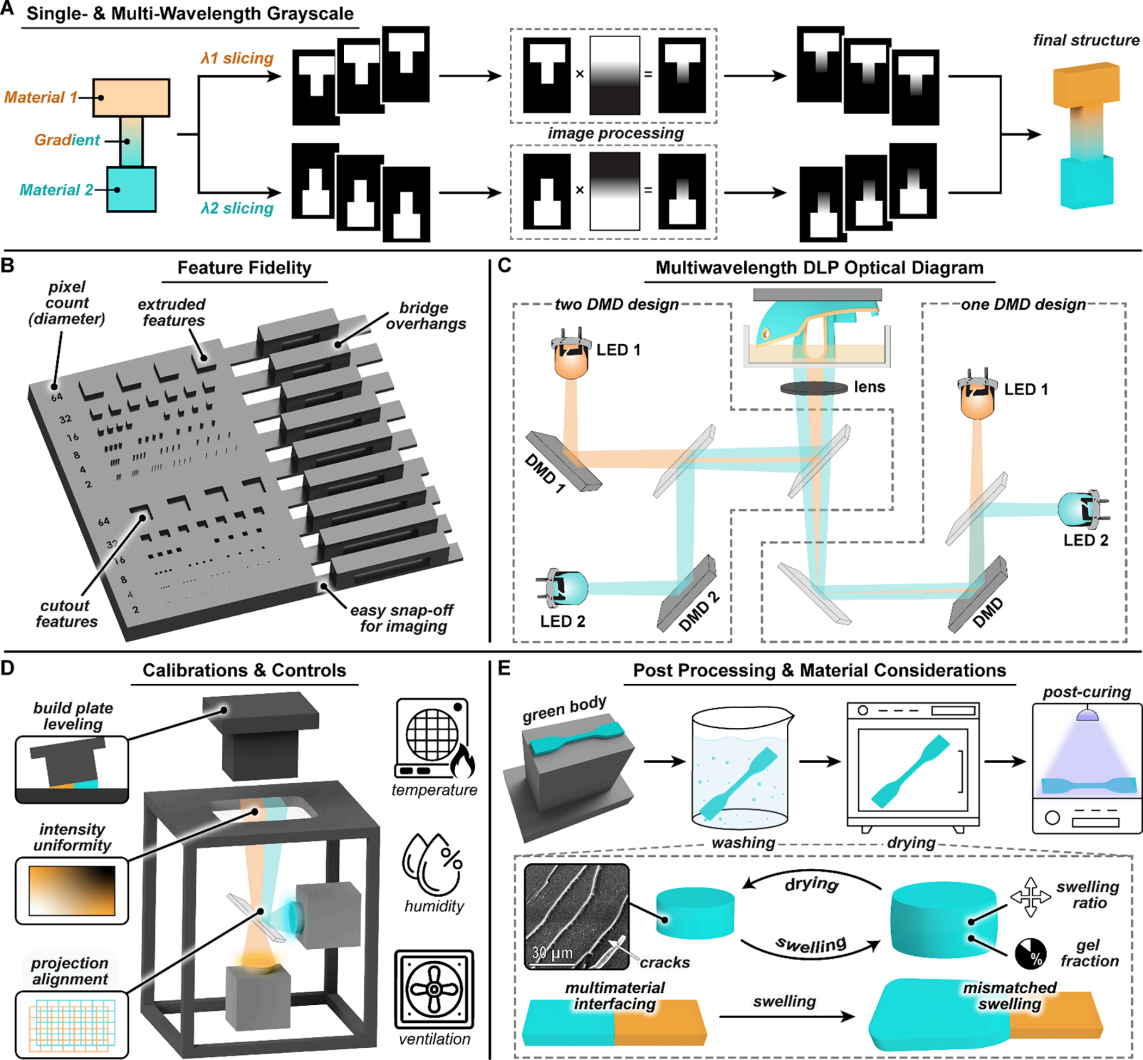
Key considerations for multimaterial DLP 3D printing using wavelength-selective resins. (A) Grayscale image processing workflow for single- and multiwavelength exposure. (B) 3D file with overhangs, extrusions, and cutouts designed for analyzing X, Y, and Z resolution in a single print. (C) Optical layouts for single- and dual-DMD printer designs, enabling independent light control. (D) Calibration and environmental controls for multimaterial VPP. (E) Postprocessing steps (washing, drying, postcuring) and key material factors of swelling ratio and gel fraction that impact print quality.

#### 2.2.2 Print Fidelity

Print fidelity in VPP can be assessed by resolving the smallest features, with pegs and holes used for lateral resolution and overhangs for vertical resolution. To support benchmarking, we provide a standardized test file (Figure 3B).^55^ Feature accuracy in single-material systems is typically improved by adjusting exposure parameters, incorporating opaquing agents,^28^ and tuning resin viscosity to limit diffusion. Inhibitors such as TEMPO or acid/base buffers further enhance spatial control in radical and ionic systems, respectively. These strategies extend to multimaterial printing, though additional considerations, like wavelength-specific absorption and selective reactivity, must be addressed. Reproducibility also depends on careful printer calibration and environmental control, including platform leveling, light uniformity, and projection alignment in dual DMD systems (Figure 3C). Consistently monitoring temperature, humidity, and ventilation further supports reliable printing outcomes and ensures user safety (Figure 3D). Looking ahead, the development of standardized calibration protocols and validation tools will be essential for enabling reproducible fabrication and accelerating the broader adoption of multimaterial VPP.

#### 2.2.3 Postprint Processing

Postprocessing steps, typically including washing, drying, and post curing, are essential for improving the mechanical integrity and aging resistance of printed parts. However, they can also distort geometry or introduce defects such as voids and stress concentrations. These effects are especially pronounced in multimaterial structures, where differences in swelling behavior and gel fraction between domains can lead to strain-induced deformation or interfacial stress during solvent removal and drying (Figure 3E). Quantifying swelling ratios and sol:gel fractions provides useful insight into these behaviors. Moving forward, a more systematic understanding of how postprocessing impacts multimaterial interfaces will be key to developing strategies that minimize defects and preserve part performance.

### 2.3 Prong III: Thermomechanical Characterization

*How can the research community develop—and more consistently apply—rigorous standardized methods to assess the thermomechanical properties of multimaterial 3D printed structures?*

A crucial first step is to characterize each material independently to establish property benchmarks. Reproducible testing then requires standardized methods along with careful attention to sample geometry, print orientation, and domain-specific behavior. While ASTM protocols offer a foundation, multimaterial systems present unique challenges that remain underexplored. Advancing thermomechanical analysis in one-pot multimaterial VPP will therefore depend on progress across four key areas: mechanical characterization (Section 2.3.1), thermal analysis (Section 2.3.2), stability testing (Section 2.3.3), and strategies for probing material interfaces (Section 2.3.4).

Quantifying print quality goes beyond dimensional accuracy to encompass thermal and mechanical performance, which become increasingly complex in multimaterial structures, particularly at interfaces prone to failure.

#### 2.3.1 Mechanical Characterization

Standardized mechanical testing is essential for comparing 3D printed materials, with unified geometries improving reproducibility across laboratories (Figure 4A). Uniaxial tensile testing using dogbone-shaped specimens (ASTM D638 for plastics, D412 for elastomers) is preferred for localizing strain and minimizing grip failure. Given the potentially anisotropic nature of VPP-printed parts, specimens should be printed in multiple orientations for testing. Compression (ASTM D695/D395) and bending (ASTM D790/D1052) tests provide additional insight into material behavior under different loading modes, supporting application-driven material selection. Hardness (ASTM D2240) is qualitative and should supplement—not replace—more rigorous tests.

**Figure 4:**
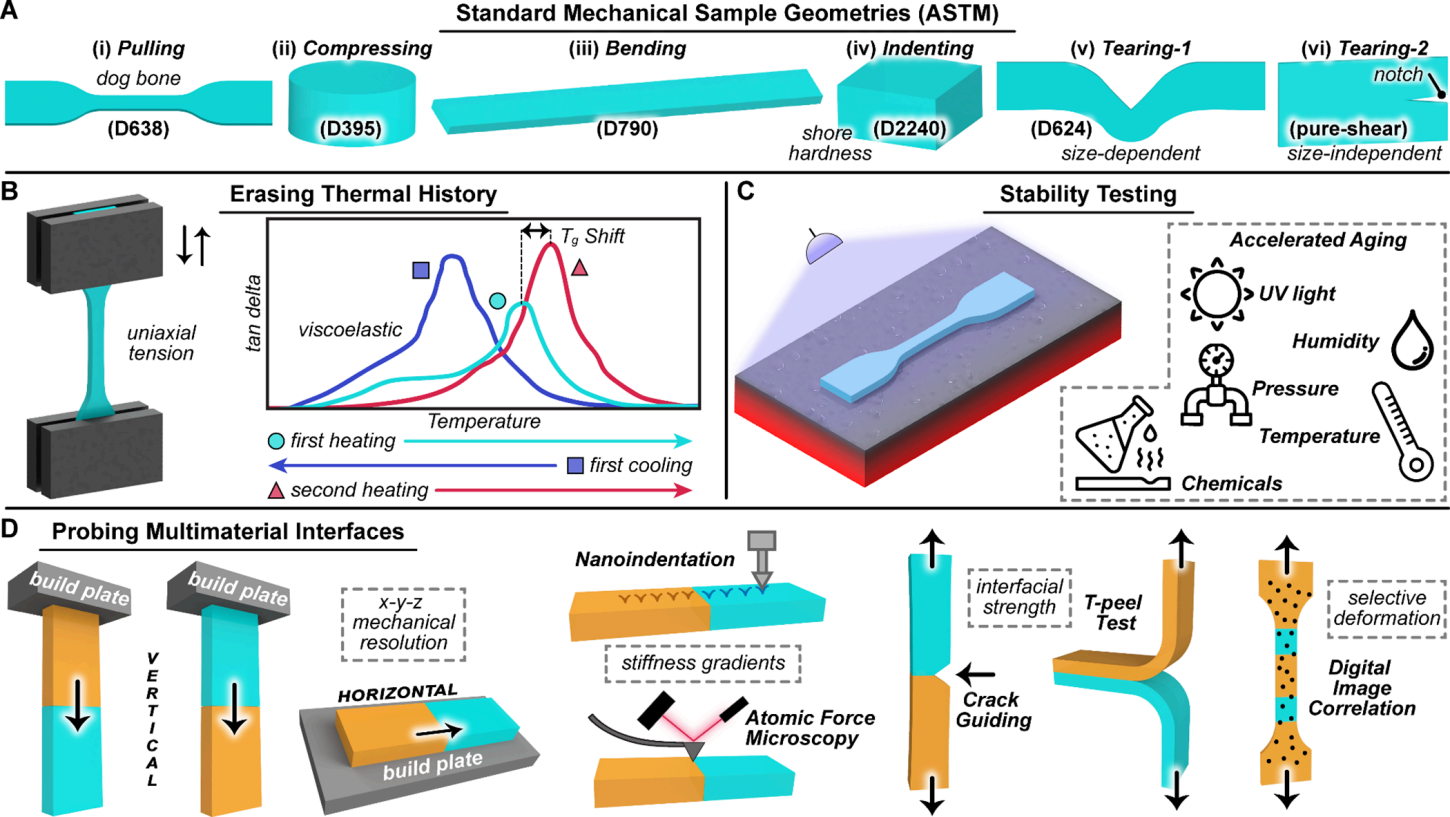
Characterization strategies for multimaterial printed parts. (A) Representative ASTM standard test geometries used to evaluate mechanical properties. (B) Thermal history assessment done using dynamic mechanical analysis (DMA). (C) Accelerated stability testing to assess long-term aging of 3D printed samples upon exposure to UV light, humidity, pressure, elevated temperature, and chemicals. (D) Multimaterial interface performance characterized via tensile testing, nanoindentation, atomic force microscopy, notch tests, T-peel tests, and digital image correlation for spatial mapping of mechanical behavior.

The term *toughness* is often used without a clear definition, though it may refer to either strain energy density (modulus of toughness) or fracture toughness from crack propagation in notched samples. Toughness metrics and test methods must be clearly defined for comparability. While ASTM D624 and related tear tests are available, their geometry dependence limits generality; planar tension (pure shear) geometries offer a more reliable, size-independent alternative.^56–58^ In addition to static tests, dynamic mechanical analysis provides key insights into viscoelasticity and durability. Hysteresis (ASTM D945 and D5992), fatigue (ASTM D7791, D4482, and D575), and creep (ASTM D2990) are particularly relevant for multimaterial structures, where interfacial mismatch can lead to premature failure. Overall, we recommend standardized tensile (ASTM D638/D412), compression (ASTM D695/D395), bending (ASTM D790/D1052), hardness (ASTM D2240), and dynamic tests (e.g., ASTM D7791, D2990) be adopted consistently across multimaterial VPP systems to enable reliable benchmarking and interlaboratory comparisons. Moving forward, expanded use of standardized, multimodal mechanical testing will be crucial for evaluating and optimizing performance in complex printed systems.

#### 2.3.2 Thermal Analysis

Thermal properties play a critical role in defining the application range of 3D printed materials, yet standardized protocols for their evaluation remain limited compared with mechanical testing (Figure 4B). Common techniques include thermogravimetric analysis (TGA), differential scanning calorimetry (DSC), and dynamic mechanical analysis (DMA). While DSC typically employs a heat–cool–heat cycle to erase thermal history, this approach is seldom applied in DMA. We recommend thermal cycling in DMA to separate kinetically trapped properties (first heating) from equilibrated behavior (second heating). In addition, in situ thermal stability can be assessed by tracking storage modulus during stepwise DMA isotherms. Overall, we recommend consistent use of standardized methods including DSC (ASTM D3418), TGA (ASTM E1131), and DMA in tensile mode (ASTM D4065), with detailed reporting of measurement rates, sample mass, atmosphere, and thermal cycling conditions to ensure reproducibility and comparability across multimaterial VPP systems.

#### 2.3.3 Stability Testing

Stability and aging resistance are rarely reported for 3D printed materials, multimaterial or otherwise, despite the availability of relevant ASTM standards developed for photocurable coatings (Figure 4C). Protocols such as ASTM G154 simulate environmental aging through controlled UV exposure to predict long-term weathering performance,^59^ while ASTM D648 and D573 assess thermal aging under elevated temperatures. For biomedical applications, parts should also be tested under sterilization conditions involving heat, pressure, and humidity.^60^ Additionally, chemical resistance for applications in harsh environments, such as exposure to solvents and environmental elements, remains an important yet often overlooked factor.^61^ Moving forward, incorporating these assessments will be important to qualify materials intended for use in harsh, long-duration, or application-specific service conditions.

#### 2.3.4 Probing Interfaces

In multimaterial 3D printed structures, interfaces often dictate mechanical performance yet remain undercharacterized (Figure 4D). Existing methods—such as uniaxial tensile and peel tests across material boundaries—provide bulk insights but lack spatial resolution.^62–64^ Techniques like nanoindentation and atomic force microscopy (AFM)^65–67^ offer micron- and submicron-scale mechanical mapping, respectively, though AFM is limited to small imaging areas. Voxel-level mechanical characterization will be key to connecting processing, reaction kinetics, and local properties. Adapting methods from 2D material studies, such as interfacial notch testing, may also provide a path toward quantifying interfacial toughness. Progress will depend on strategies to measure thermomechanical behavior with sufficient resolution for multimaterial evaluation.

## 3 Concluding Remarks

Multimaterial VPP 3D printing holds tremendous promise for fabricating complex functional structures by uniting advances in resin chemistry, light-mediated processing, and precision characterization. Unlocking this potential will require expanding beyond conventional acrylic-based systems through improved control over the reaction selectivity, wavelength responsiveness, and resin compatibility. Hardware innovations, such as high-intensity, narrow-bandwidth light sources, and software capable of grayscale and multiwavelength modulation will be essential for achieving spatially defined material placement. At the same time, rigorous and standardized approaches to thermomechanical testing along with emerging tools for voxel-level interface characterization will be critical for ensuring performance and reproducibility. While this Outlook focused on experimental approaches, we anticipate that computational modeling will also play a crucial role in guiding the design of multimaterial structures, much like topology optimization has done for single-material systems. Defining clear application targets, whether in robotics, medicine, aerospace, or beyond, will help focus innovation on structures with graded compositions and engineered interfaces tailored for optimal performance.

To fully realize the impact of these advances, researchers must move beyond monolithic design paradigms and embrace strategies centered on spatially programmable multimaterial integration.

## Supplementary Material



## Notes

Transparent Peer Review report available (PDF)

## §

School of Chemical Engineering and Applied Chemistry, Department of Chemical Engineering, Kyungpook National University, 80 Daehak-ro, Buk-gu, Daegu 41566, Republic of Korea
