# Supplementary material for: One-Vat Multimaterial 3D Printing: The Devil is in the Details
Source: ACS Cent Sci. 2025 Aug 7;11(9):1527–35. doi: 10.1021/acscentsci.5c00986 (PMC12464769; doi:10.1021/acscentsci.5c00986)
Supplement: Supplementary file 1 [file oc5c00986_si_001.pdf]

oc-2025-00986x.R1

Name: Peer Review Information for "One-Vat Multimaterial 3D Printing: The Devil is in the Details"

First Round of Reviewer Comments

Reviewer: 1

Comments to the Author

The manuscript provides an overview of recent advances and challenges in single-vat multimaterial vat photopolymerization (VPP) 3D printing, emphasizing the integration of multiple materials within a single structure to achieve properties unachievable by monolithic systems. This manuscript serves as useful overview for the beginning researcher in the AM field including chemical platforms and analytical tools to demonstrate success. The review is structured around three main "prongs" as defined from the authors, although another term might be considered. The first is resin formulation and photochemistry, highlighting the need for new chemistries and more thorough photochemical characterization. The second is printing strategies, focusing on grayscale and multiwavelength light control to spatially program material properties. The third is thermomechanical characterization, stressing the importance of standardized testing to ensure reproducibility and reliability of printed multimaterial structures. An important section is the aging and durability section, which could benefit from more depth since such a key emerging concern for AM parts. Key findings include the identification of current limitations in resin diversity, challenges in reaction selectivity, and the need for improved calibration and post-processing protocols. The manuscript also emphasizes the critical role of rigorous, standardized mechanical and thermal testing, as well as the development of new tools for probing material interfaces at high spatial resolution. The manuscript requires minor revisions, primarily editing and some additional depth, before being considered for publication in ACS Central Science.

Comments:

- The author must cite every claim; significant amounts of claims are uncited (e.g. "multimaterial printing enables functional variation across mechanical, thermal, optical,

and electrical properties" and "most printed parts still use only one material"). A more thorough citation attention would add to the value and impact of this review.

- The author must ensure titles and headers are cohesive and consistent throughout the manuscript, with section 2.1 matching the format and structure of sections 2.2 and 2.3, and all section headers aligned with those in the introduction. This will result in a more readable manuscript.

- The figures are very complex but exceptionally well done, and if simplified somewhat then the readers could use the figures with permission more liberally.

- The author must ensure that all figure captions use terminology consistent with the graphics and revise figures to avoid confusion or clutter.

- The author must ensure all word choices are uniformly used throughout the paper (e.g. "thermomechanical" versus "thermo-mechanical"). In addition, In-fact, with a hyphen is awkward.

- The author must proofread the manuscript carefully for grammar.

- The author should discuss limitations of current photochemical mechanisms in more detail and suggest specific future research directions for multimaterial VPP. The authors might also refer to multimodality printing and multi-vat printing, which has received renewed attention.

- The author should clarify the distinction between mechanical and thermal characterization methods and provide explicit standardization recommendations.

- The author should consider including a table of recommended standardized testing protocols for multimaterial VPP for clarity. The inclusion of ASTM methods was very useful, but the Table would make this easier for the reader to access.

- The author should discuss the potential impact of computational modeling in guiding experimental design and integrating with multimaterial VPP. This would be a nice complement to the existing organization.

Reviewer: 2

Comments to the Author

The manuscript by Page and coworkers presents an insightful and well-structured overview of emerging strategies for single-vat multimaterial Vat Photopolymerization (VPP). The authors do an excellent job of highlighting the potential of multimaterial VPP to achieve complex structures with tailored properties. The paper clearly presents the opportunities associated with advancing resin chemistry, improving reaction selectivity, and incorporating grayscale and multiwavelength light control into the process.

The manuscript is logically organized, addressing key areas such as resin formulation, printing strategies, and thermomechanical characterization, all of which are vital for advancing multimaterial VPP. The discussion of expanding resin chemistry beyond standard acrylates and the need for standardized testing methods further strengthens the article's relevance to the field.

Priot to publication, I suggest to improve the following issues:

Suggestions for Improvement:

1) One significant VAT technique that could further enhance the manuscript is the inclusion of multiphoton laser printing as a technique for multi-material fabrication. This technology allows for precise control over material deposition and can enable higher resolution and greater material contrast. Including a brief discussion of this technique could provide readers with a more comprehensive view of the current possibilities in multimaterial VPP.

2) While Figure 1 is informative, it is somewhat crowded, which could hinder clarity. Specifically, Panel A presents a high level of detail, which might overwhelm readers given that similar details are expanded upon in later figures. Simplifying Panel A would improve the figure's clarity and help focus the reader's attention on the most essential elements.

Author's Response to Peer Review Comments:

Reviewer: 1
Recommendation: Publish in ACS Central Science after minor revisions noted.

Comments:
The manuscript provides an overview of recent advances and challenges in single-vat multimaterial vat photopolymerization (VPP) 3D printing, emphasizing the integration of multiple materials within a single structure to achieve properties unachievable by monolithic systems. This manuscript serves

as useful overview for the beginning researcher in the AM field including chemical platforms and analytical tools to demonstrate success. The review is structured around three main "prongs" as defined from the authors, although another term might be considered. The first is resin formulation and photochemistry, highlighting the need for new chemistries and more thorough photochemical characterization. The second is printing strategies, focusing on grayscale and multiwavelength light control to spatially program material properties. The third is thermomechanical characterization, stressing the importance of standardized testing to ensure reproducibility and reliability of printed multimaterial structures. An important section is the aging and durability section, which could benefit from more depth since such a key emerging concern for AM parts. Key findings include the identification of current limitations in resin diversity, challenges in reaction selectivity, and the need for improved calibration and post-processing protocols. The manuscript also emphasizes the critical role of rigorous, standardized mechanical and thermal testing, as well as the development of new tools for probing material interfaces at high spatial resolution. The manuscript requires minor revisions, primarily editing and some additional depth, before being considered for publication in ACS Central Science.

We thank the reviewer for their thoughtful and constructive feedback. We are pleased that the manuscript was found to be a useful overview for researchers in the additive manufacturing field and that the reviewer appreciated our framing around key challenges in multimaterial vat photopolymerization. Regarding the suggestion to consider a different term than "prongs," we appreciate this input. However, we have chosen to retain the term "prongs" as it aligns with the overarching metaphor used in the manuscript's title and Table of Contents graphic. We also use this imagery in Figure 1A. Our intention was to convey a three-pronged approach to addressing multimaterial VPP challenges, and we hope that with this context, the term feels more purposeful and integrated.

In response to the comment on the aging and stability section, we have included additional relevant literature with a focus on stability testing of polymeric 3D printing systems. We agree that this is an important emerging concern. While we are limited in space by the Outlook format, we have added references and a sentence to broaden the scope of the stability section to include chemically demanding environments. Specifically, we now note: "*Additionally, chemical resistance for applications in harsh environments, such as exposure to solvents and environmental elements, remains an important yet often overlooked factor.*[61]" We hope this addition strengthens the section and highlights the importance of application-specific stability testing.

Below, we address each of the reviewer's suggestions in detail and have made corresponding revisions to the manuscript where appropriate.

Comments:

- The author must cite every claim; significant amounts of claims are uncited (e.g. "multimaterial printing enables functional variation across mechanical, thermal, optical, and electrical properties" and "most printed parts still use only one material"). A more thorough citation attention would add to the value and impact of this review.

We appreciate the reviewer's careful reading and thoughtful perspective. We have revised the manuscript accordingly to add relevant citations for these claims.

- The author must ensure titles and headers are cohesive and consistent throughout the manuscript, with section 2.1 matching the format and structure of sections 2.2 and 2.3, and all section headers aligned with those in the introduction. This will result in a more readable manuscript.

We thank the reviewer for this important observation. Each heading throughout section 2 was changed to match the introduction outline. The sections now read "*Prong I: Resin Formulation*", "*Prong II: Printing Strategies*", and "*Prong III: Thermomechanical Characterization*".

- The figures are very complex but exceptionally well done, and if simplified somewhat then the readers could use the figures with permission more liberally.

We appreciate the reviewer's kind words about the quality of the figures. Figure 1A was edited to improve its clarity by introducing arrows and removing the symbols for chain-growth and step-growth. We agree that clarity is essential, but we have chosen to retain the current level of detail for the other figures to accurately convey the layered challenges and strategies discussed in the manuscript. These figures are intentionally designed as reference-style visuals that readers can return to for depth, and we believe further simplification would reduce their utility in this context. We hope they will still serve as a useful resource for the community. Here is the updated version of Figure 1A:

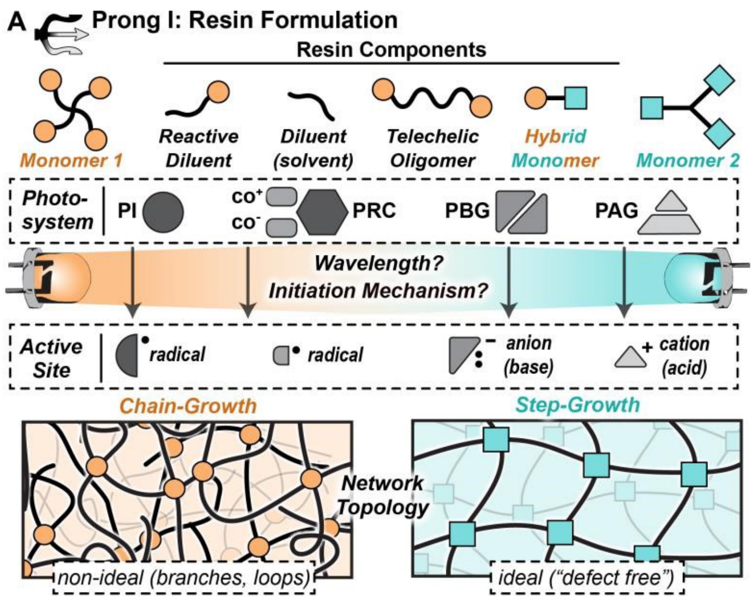

- The author must ensure that all figure captions use terminology consistent with the graphics and revise figures to avoid confusion or clutter.

We thank the reviewer for this important observation. Each heading throughout Figure 1 was changed to match the text and introduction. The sections now read "*Prong I: Resin Formulation*", "*Prong II: Printing Strategies*", and "*Prong III: Thermomechanical Characterization*". We have carefully reviewed the remaining text for consistency and clarity.

- The author must ensure all word choices are uniformly used throughout the paper (e.g. "thermomechanical" versus "thermo-mechanical"). In addition, In-fact, with a hyphen is awkward.

We appreciate the reviewer's comment and have made this correction to ensure all instances of this term is now changed to "thermomechanical". We also removed the hyphen from "Infact".

- The author must proofread the manuscript carefully for grammar.

We sincerely appreciate the reviewer's time in carefully reading our manuscript and we have completed additional proofreading and grammar corrections.

- The author should discuss limitations of current photochemical mechanisms in more detail and suggest specific future research directions for multimaterial VPP.

We are grateful for the reviewer's suggestion to expand on this point. While we agree that additional detail would be valuable, we are constrained by the space limitations of the Outlook format and have prioritized content that aligns most directly with our central themes.

Regarding the request for additional detail into limitations of current photochemical mechanisms we wanted to highlight the following text for reference:

Page 7: *"Critically, few photosystems combine rapid curing with wavelength selectivity, highlighting a pressing need for new photochemical platforms tailored for multimaterial printing."*

We think that a deeper exploration of these limitations, along with proposed future directions, would merit a dedicated review or research article and is therefore beyond the intended scope of this Outlook.

While not consolidated into a single section of our manuscript, we chose to talk about specific future directions in each section as it pertained to that topic. We wanted to highlight the following text for reference:

Page 6: "*Future work should focus on deconvoluting overlapping IR signatures – such as those between acrylates and epoxides – to better resolve reaction selectivity in complex resin systems.*

Notably, techniques like photo-DSC,[26] photo-Raman,[27,28] and photo-NMR[29] spectroscopy offer complementary insights into competitive reaction pathways."

Page 8: "*Complementing these efforts, one-electron (radical) mechanisms beyond acrylic chaingrowth polymerization offer an underexplored route to functional and potentially recyclable materials.*"

Page 9: "*Moving forward, optimized irradiation protocols and integrated software tools that encode grayscale and wavelength-specific data prior to slicing will be essential. There is also an opportunity to develop intuitive design platforms that recommend exposure strategies based on part geometry, material requirements, and intended functionality."*

Page 10: "*Looking ahead, the development of standardized calibration protocols and validation tools will be essential for enabling reproducible fabrication and accelerating broader adoption of multimaterial VPP.*"

Page 10: "*Moving forward, a more systematic understanding of how post processing impacts multimaterial interfaces will be key to developing strategies that minimize defects and preserve part performance.*"

- The authors might also refer to multimodality printing and multi-vat printing, which has received renewed attention.

We value the reviewer's recommendation to elaborate further, and we have done so accordingly. Within the introduction under Prong II: Printing Strategies, we discuss multi-vat printing and have added multimodality printing as well. This section now reads: "*One avenue toward multimaterial 3D printing is multimodality printers, which combine multiple 3D printing techniques into a single system; however, this approach requires highly specialized hardware and custom software integration.[8,9].... However, current multimaterial VPP is largely multi-vat based, which slows printing and risks cross-contamination.[11]"*

- The author should clarify the distinction between mechanical and thermal characterization methods and provide explicit standardization recommendations.

We thank the reviewer for this helpful suggestion. In response, we have revised the relevant sections to more clearly distinguish between mechanical and thermal characterization methods. We also added additional specific ASTM standard reference numbers under the thermal analysis section.

Under the mechanical characterization section, we have added this sentence (page 12): *"Overall, we recommend standardized tensile (ASTM D638/D412), compression (ASTM D695/D395), bending (ASTM D790/D1052), hardness (ASTM D2240), and dynamic tests (e.g., ASTM D7791, D2990) be adopted consistently across multimaterial VPP systems to enable reliable benchmarking and interlaboratory comparisons."*

Under the thermal analysis section, we have added this sentence (page 13): "*Overall, we recommend consistent use of standardized methods including DSC (ASTM D3418), TGA (ASTM*

E1131), and DMA in tensile mode (ASTM D4065), with detailed reporting of scan rates, sample mass, atmosphere, and thermal cycling conditions to ensure reproducibility and comparability across multimaterial VPP systems."

- The author should consider including a table of recommended standardized testing protocols for multimaterial VPP for clarity. The inclusion of ASTM methods was very useful, but the Table would make this easier for the reader to access.

We appreciate the reviewer's suggestion to include a summary table of standardized testing protocols. We agree that such a table could enhance accessibility for readers. However, due to the space limitations of the Outlook format for *ACS Central Science*, we are unable to include an additional table without removing other key content. To address the reviewer's suggestion within the available space, we have revised the relevant sections (mentioned above) to more clearly highlight the ASTM protocols in the text and improved their visibility by summarizing our explicit recommendations at the end of each section. We hope this provides the intended clarity while maintaining alignment with journal formatting constraints.

- The author should discuss the potential impact of computational modeling in guiding experimental design and integrating with multimaterial VPP. This would be a nice complement to the existing organization.

We thank the reviewer for this insightful suggestion. While we agree that computational modeling will be an important tool for advancing multimaterial VPP, this Outlook was intentionally focused on experimental strategies. We do briefly acknowledge the relevance of modeling on Page 13: "*While this Outlook focused on experimental approaches, we anticipate that computational modeling will also play a crucial role in guiding the design of multimaterial structures – much like topology optimization has done for single-material systems*."

Additionally, as computational modeling lies outside our core area of expertise, we think that a more in-depth discussion would be better suited to a separate perspective or review article authored by experts in this field.

Reviewer: 2
Recommendation: Publish in ACS Central Science after minor revisions noted.

Comments:
The manuscript by Page and coworkers presents an insightful and well-structured overview of emerging strategies for single-vat multimaterial Vat Photopolymerization (VPP). The authors do an excellent job of highlighting the potential of multimaterial VPP to achieve complex structures with tailored properties. The paper clearly presents the opportunities associated with advancing resin chemistry, improving reaction selectivity, and incorporating grayscale and multiwavelength light control into the process.

The manuscript is logically organized, addressing key areas such as resin formulation, printing strategies, and thermomechanical characterization, all of which are vital for advancing multimaterial VPP. The discussion of expanding resin chemistry beyond standard acrylates and the need for standardized testing methods further strengthens the article's relevance to the field. Priot to publication, I suggest to improve the following issues:

We sincerely thank the reviewer for their thoughtful and encouraging assessment of our manuscript. We are pleased to hear that the overall structure, clarity, and relevance of the work were well received. Below, we address each of the reviewer's suggestions in detail and have made corresponding revisions to the manuscript where appropriate.

Suggestions for Improvement:

1) One significant VAT technique that could further enhance the manuscript is the inclusion of multiphoton laser printing as a technique for multi-material fabrication. This technology allows for precise control over material deposition and can enable higher resolution and greater material contrast. Including a brief discussion of this technique could provide readers with a more comprehensive view of the current possibilities in multimaterial VPP.

We thank the reviewer for highlighting multiphoton laser printing as a valuable technique for multimaterial fabrication. Given the scope and space constraints of this Outlook, we have chosen to focus more extensively on vat photopolymerization methods with broader accessibility and scalability, such as digital light processing (DLP). However, we appreciate the suggestion and have ensured the existing mention of MPP reflects its unique advantages and challenges in multimaterial VPP.

We have added this sentence along with two additional references within the introduction under *Prong II: Printing Strategies* (page 2): *"On the other end of the spectrum, multi-photon polymerization (MPP) enables submicron resolution, but it remains limited by low throughput, high equipment costs, and poor scalability.[6,7]"*

2) While Figure 1 is informative, it is somewhat crowded, which could hinder clarity. Specifically, Panel A presents a high level of detail, which might overwhelm readers given that similar details are expanded upon in later figures. Simplifying Panel A would improve the figure's clarity and help focus the reader's attention on the most essential elements.

We appreciate the reviewer's thoughtful suggestion. In response, we have edited Figure 1A to improve clarity by removing non-essential text and adding directional arrows to better illustrate the logical flow of the framework. We hope these refinements improve readability while preserving the intended level of detail for contextual understanding. Here is the updated version of Figure 1A:

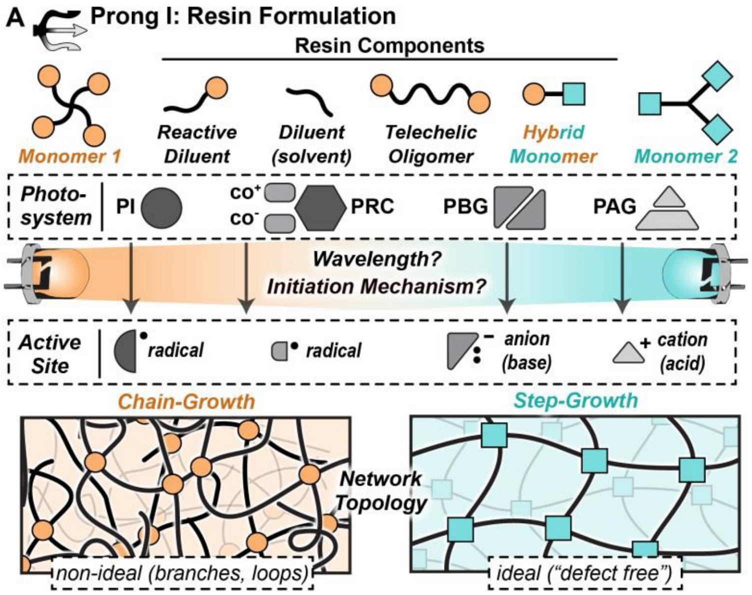
A
Prong I: Resin Formulation
Resin Components
Monomer 1
Reactive Diluent
Diluent (solvent)
Telechelic Oligomer
Hybrid Monomer
Monomer 2
Photo-system
PI
co+
co-
PRC
PBG
PAG
Wavelength?
Initiation Mechanism?
Active Site
radical
radical
anion (base)
cation (acid)
Chain-Growth
Step-Growth
Network Topology
non-ideal (branches, loops)
ideal ("defect free")
